# Open Communication and Discussion Facilitate Reconciliation: The Maimonides Approach to Publication of Manduca’s Letter in *The Lancet*

**DOI:** 10.5041/RMMJ.10176

**Published:** 2015-01-29

**Authors:** Shraga Blazer

**Affiliations:** Editor-in-Chief, Rambam Maimonides Medical Journal and Director of the Department of Neonatology and Neonatal Intensive Care Unit, Rambam Health Care Campus, Haifa, Israel

Dear Friends and Colleagues,

In August of 2014, Manduca P et al. published “An open letter for the people in Gaza” in *The Lancet*. This letter was the response of those authors to their perspective of what was happening in Gaza during the summer-long conflict between Israel and Gaza. Israel was finally responding to years of bombardment from Gaza into civilian areas in the south of Israel. Two of the authors of the letters were known anti-Semites, and held connections with David Duke, a former Ku Klux Klan Grand Wizard in Louisiana and advocate of Nazism. Both these authors expressed sympathy and support for Duke’s rabidly anti-Jewish positions. In their letter they accused Israel’s medical community of complicity in committing terrible atrocities and even implied that chemical warfare was being used by Israel.

The publication of that letter in *The Lancet* created a storm that extended far beyond the scientific academic world and the Jewish community. At the heart of the issue was the question, “How could a scientific medical journal become involved with political issues?” In addition, if such a letter or article was to be publicized, why wasn’t the information scrutinized, and why wasn’t the perspective of those accused of atrocities sought? The general response in the academic world, particularly among Jewish colleagues, was an angry call to boycott *The Lancet*, which already had a long anti-Israeli reputation. In addition, people were demanding that the Editor-in-Chief of *The Lancet* should be replaced.

From the perspective of Rambam Health Care Campus, observing the situation made it obvious that the Editor-in-Chief of *The Lancet* (Dr. Richard Horton), was unfamiliar with the health and medical aspects of Israel at many levels. An offensive approach in personal relationships is never constructive. Hence, it was felt that rather than joining the masses in anger, we should extend an invitation to someone who might not be familiar with the delicate structure of Israeli healthcare and its relationship to the local Israeli Arabs, as well as our neighbors. Professor Karl Skorecki, Director of Medical and Research Development at Rambam, with the full support of Professor Rafi Beyar, Director of Rambam, invited Dr. Horton to visit Rambam Health Care Campus and communities nearby, as mirrors of the Israeli healthcare system, “because of the intense interest that the editorial leadership of *The Lancet* has attracted, focusing on issues of medical professional responsibility and accountability for the tragic loss of life and human suffering of Gaza civilians including children.”

Dr. Horton graciously and courageously accepted that invitation. On October 24, 2014, Rambam Health Care Campus hosted him for four days. Dr. Horton met with some of Rambam’s diverse staff, including Jewish and Arab Israeli physicians and scientists who maintain respectful relationships in and out of work. He saw for himself Arab and Jewish health care professionals working hand-in-hand caring for needy patients from diverse backgrounds and communities from Israel, Gaza, the West Bank, and even Syria—without discrimination—providing the same high standards of compassionate care to each patient. In addition, Dr. Horton attended medical and scientific lectures presented by Rambam physicians (Jewish and Arab), and participated in a debate that focused on issues related to the publication of the controversial letter in *The Lancet*. Dr. Horton’s visit culminated in a Rambam Grand Rounds lecture focusing on the “Geopolitical Issues and Responsibilities of Medical and Scientific Journals.”

At the opening of the Grand Rounds Lecture, Dr. Horton said that he deeply regretted the “unnecessary polarization that publication of the letter of Paola Manduca caused.” Throughout his lecture, he offered a hand of partnership to the Israeli medical community and concluded with an offer to partner with Israeli medical professionals to produce a series of publications in *The Lancet* on Israeli health, healthcare, and medical research. In addition, he subsequently publicized new recommendations for publication of editorial positions in *The Lancet*.

In the spirit of the teachings of Maimonides, the editors of *Rambam Maimonides Medical Journal* felt that highlights of Dr. Horton’s transformative visit should be made available to the scientific and medical community worldwide. Hence, we decided to provide an edited transcription of a debate held at Rambam focusing on the place of politics in medical scientific publications, and a transcription and link to the video of Dr. Horton’s Rambam Grand Rounds lecture. Our hope is that publication of the debate and the video will help to contribute to this new bridge-building effort on the part of *The Lancet* to present the scientific medical world of Israel in a more balanced light.

Having met personally with Dr. Horton, I was deeply impressed by his sincerity. He is a warm individual, extremely serious, with a strong belief that health care can and should be a unifying force. In the course of our meetings, it was clear that prior to his visit, Dr. Horton’s knowledge of Israel and its many peoples was incomplete, and he learned about the unique challenges facing the healthcare system in Israel. Contrary to those who question Dr. Horton’s intent and efforts, I am personally convinced that only good for the people of Israel and her neighbors will emerge from his visits—indeed, Dr. Horton visited Israel and Rambam again just a few days before publication of this issue. He attended several meetings throughout Israel, and began working with Israeli colleagues on the articles to be published in *The Lancet*.

I look forward to future collaborations that will bring Israel into the scientific and medical world of *The Lancet* and beyond.

Shraga Blazer, MDEditor-in-ChiefRambam Maimonides Medical Journal

